# Successful surgical closure of infected abdominal wounds following preconditioning with negative pressure wound therapy

**DOI:** 10.1007/s00423-021-02221-w

**Published:** 2021-06-17

**Authors:** Johanna C. Wagner, Anja Wetz, Armin Wiegering, Johan F. Lock, Stefan Löb, Christoph-Thomas Germer, Ingo Klein

**Affiliations:** 1 Department of General, Visceral, Transplantation, Vascular and Pediatric Surgery, University Hospital Würzburg, Oberduerrbacherstr. 6, 97080 Würzburg, Germany; 2 Comprehensive Cancer Center Mainfranken, Würzburg, Germany

**Keywords:** Surgical site infections, Secondary skin closure, Negative pressure wound therapy, Open wound treatment

## Abstract

**Purpose:**

Traditionally, previous wound infection was considered a contraindication to secondary skin closure; however, several case reports describe successful secondary wound closure of wounds “preconditioned” with negative pressure wound therapy (NPWT). Although this has been increasingly applied in daily practice, a systematic analysis of its feasibility has not been published thus far. The aim of this study was to evaluate secondary skin closure in previously infected abdominal wounds following treatment with NPWT.

**Methods:**

Single-center retrospective analysis of patients with infected abdominal wounds treated with NPWT followed by either secondary skin closure referenced to a group receiving open wound therapy. Endpoints were wound closure rate, wound complications (such as recurrent infection or hernia), and perioperative data (such as duration of NPWT or hospitalization parameters).

**Results:**

One hundred ninety-eight patients during 2013–2016 received a secondary skin closure after NPWT and were analyzed and referenced to 67 patients in the same period with open wound treatment after NPWT. No significant difference in BMI, chronic immunosuppressive medication, or tobacco use was found between both groups. The mean duration of hospital stay was 30 days with a comparable duration in both patient groups (29 versus 33 days, *p* = 0.35). Interestingly, only 7.7% of patients after secondary skin closure developed recurrent surgical site infection and in over 80% of patients were discharged with closed wounds requiring only minimal outpatient wound care.

**Conclusion:**

Surgical skin closure following NPWT of infected abdominal wounds is a good and safe alternative to open wound treatment. It prevents lengthy outpatient wound therapy and is expected to result in a higher quality of life for patients and reduce health care costs.

## Introduction

Surgical site infections (SSI) are the most common nosocomial infection, comprising to 20.0–24.3% of all nosocomial infections [1, 2]. The overall incidence is estimated to be about 1.8–1.9% [1, 3, 4]. This results in an increase in patient morbidity and in some cases mortality, a prolonged hospital stay or re-admission, and a profound financial burden of the health care systems [2–6]. Thus, the prevention and optimal treatment of SSIs is paramount. SSIs are classified into three groups: superficial incisional SSI (skin and subcutaneous tissue), deep incisional SSI (facial and muscle layers), and organ/space SSI [7]. The recommended therapy for these SSIs differs; however, one main concept is source control. Source control in SSIs in the subcutaneous, fascial, and muscle layers requires the reopening of the skin sutures for drainage and consecutive open wound healing. This wound healing process requires daily wound dressing changes and gradual filling of the defect by granulation tissue. This is a lengthy process with a significant reduction in quality of life for the patients [8] and a considerable increase in health care costs [9].

In the late 1980s, Morykwas and Argenta developed the negative pressure wound therapy (NPWT) to treat primarily chronic and infected wounds [10]. The goal was to increase patient comfort and decrease morbidity, length of hospital stay, and health care costs [10]. The NPWT provides a negative pressure, which is applied evenly to the entire wound surface. This leads to an effective drainage of excess fluids and wound debris and increases capillary blood flow [11]. Furthermore, the NPWT increases mechanical tissue stress, and thus, promotes and accelerates tissue healing [12, 13]. The use of negative pressure therapy for wounds healing by secondary intention has increased in spite of the lack of evidence of its advantage [14]. Just recently, one randomized control trial has been published comparing negative pressure therapy to conventional wound therapy [15]. The results showed that negative pressure therapy reduces the time until complete wound closure and increases the number of wounds closed after 42 days. However, the number of wound-related adverse events was higher in the group with negative pressure therapy (periwound macerations and local infections) [15]. In many cases, wounds show a clean appearance and bacterial swabs do not show any growth after a few cycles of NPWT. Yet, there are no guidelines for subsequent measures after NPWT. Secondary skin closure of wounds managed with open wound treatment or negative pressure therapy is not considered standard of care because of potential bacterial contamination of the subcutaneous tissue and the risk of recurrent surgical site infection.

The aim of this retrospective study was to evaluate the success rate, effectiveness, and potential benefit of secondary skin closure in abdominal wounds following preconditioning treatment with NPWT in a larger consecutive cohort.

## Methods

This retrospective, single-center study was conducted at a German surgical tertiary care center. Patients hospitalized between January 2013 and March 2016 requiring NPWT after abdominal surgery and surgical site infection with subcutaneous abscess formation were included (superficial and deep incisional surgical site infections [7]). The application or dressing change of a NPWT was conducted according to the manufacturer’s guidelines [16] and previous reports [11] in a clean, but not necessarily sterile, environment. Sterile, black polyurethane sponge material was accurately fitted and placed into the wound bed. An adhesive foil was placed on top of the sponge and secured the wound dressing to normal skin. With the help of a non-collapsible tube, the sponge was connected to a vacuum pump (pressure − 25 to − 200 mmHg). We excluded patients, who were transferred to or from our institution with a NPWT in place, and thus included only those patients with the entire NPWT at our institution. As a total of 10 patients had two different surgeries during separate hospitalizations within the 3 years of investigation, we only included the first hospitalization to avoid confounding factors. Once the subcutaneous, facial, and muscular tissue clinically appeared non-infected, a secondary, surgical wound and skin closure was planned (i.e. no clinical signs of local infection, clear and small quantities of wound fluid). The secondary, surgical wound closure was done in the operating room in sterile conditions with the following steps: removal of the NPWT, wound irrigation, insertion of a suction drain, and suture using a 2–0 prolene suture. Patient characteristics, such as sex, age, body mass index (BMI), diagnosis, tobacco use, chronic medication, previous abdominal surgeries, and the ASA status (American Society of Anesthesiologists), were collected. Concerning the NPWT, the point in time of application, the duration of the therapy, and the number of dressing/sponge changes were analyzed. The following endpoints were analyzed: length of hospital stay, length of ICU (intensive care unit) and/or IMC (intermediate care unit) stay, and the type of discharge. To evaluate the long-term wound outcome, the wound condition of patients visiting the outpatient clinic was included until April 2018. During this follow-up period, the frequency and time intervals of patients visiting the outpatient clinic varied due to the retrospective nature of the study. In addition, patients without surgical wound closure and conversion to open wound therapy after NPWT were analyzed as well. Open wound therapy at our institution is performed by nurses and physicians with dressing changes daily or every other day based on the products used. For the open wound treatment, different dressing materials were used depending on the wound morphology (gauze, dialkylcarbamoylchloride-coated gauze, polyurethane-based materials).

The data was collected and analyzed with Microsoft Excel. GNU PSPP was used for the statistical analysis and GraphPad Prism 9 was used to generate the figures. The *t* test for independent samples was used for numeric data and the chi-square test for nominal data. A *p* < 0.05 was considered significant.

## Results

### Patient population and characteristics

**Fig. 1:**
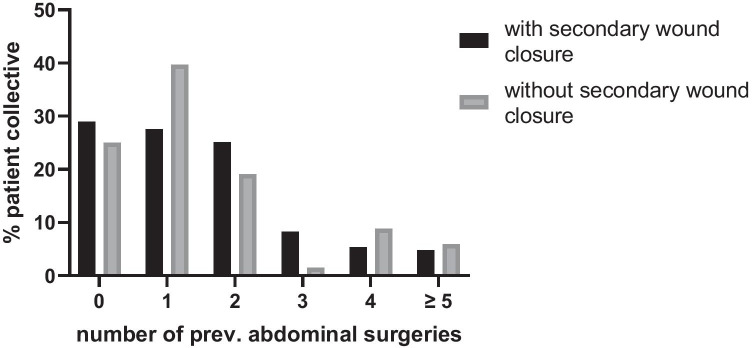
Number of previous abdominal surgeries

One hundred ninety-eight patients received a secondary skin closure after NPWT at our institution between January 2013 and March 2016. Patient characteristics are provided in Table 1. The average number of previous abdominal surgeries was 1.50 ± 1.50 (Fig. 1).

**Table 1:**
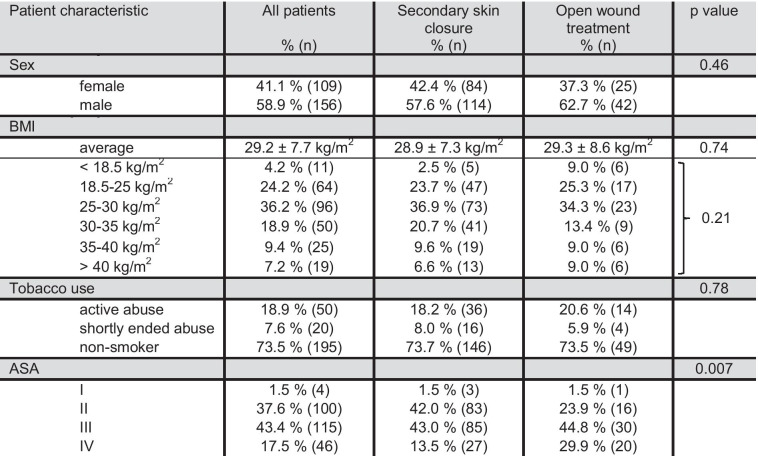
Patient characteristics

### Surgical data

We then looked at the urgency and type of surgery as well as the type of incision. The majority of wound infections occurred after emergency surgery (surgery within 12 h) (54.0%, *n* = 99). The most common reason for elective surgery was oncological surgeries. In the group of patients receiving emergency surgery, however, the indication for surgery was more diverse (Table 2). The most common incision type was a midline laparotomy (55.1%, *n* = 109), followed by a transverse laparotomy (14.1%, *n* = 28).

**Table 2:** Urgency and indication for surgery compared between the patients with and without secondary skin closure

Urgency and indication for surgery	With secondary skin closure (*n*)	Without secondary skin closure (*n*)
Emergency	54.0% (99)	52.2% (35)
Wound healing disorder	18.2% (18)	17.1% (6)
Intestinal perforation	15.2% (15)	14.3% (5)
Abscess	15.2% (152)	2.9% (1)
Cholecystitis	7.1% (7)	2.9% (1)
Intestinal ischemia	6.1% (6)	17.1% (6)
Infection of implant	6.1% (6)	0%
Hematoma	6.1% (6)	0%
Anastomotic leak	5.1% (5)	11.4% (4)
Ileus	5.1% (5)	11.4% (4)
Incarcerated hernia	5.1% (5)	2.9% (1)
Polytrauma	2.0% (2)	5.7% (2)
Intestinal stenosis	1.0% (1)	8.6% (3)
other	8.1% (8)	5.7% (2)
Elective	39.9% (78)	37.3% (25)
Carcinoma	60.3% (47)	40.0% (10)
Inflammatory bowel disease	10.3% (8)	0%
Chronic soft tissue infection	6.4% (5)	8.0% (2)
Ostomy reversal	7.7% (6)	4.0% (1)
Hernia	5.1% (4)	36.0% (9)
Intestinal stenosis	3.8% (3)	4.0% (1)
Liver transplantation	3.8% (3)	0%
Sigma diverticulitis	2.5% (2)	8.0% (2)
Unknown	10.1% (21)	10.3% (7)

### NPWT data

**Fig. 2:**
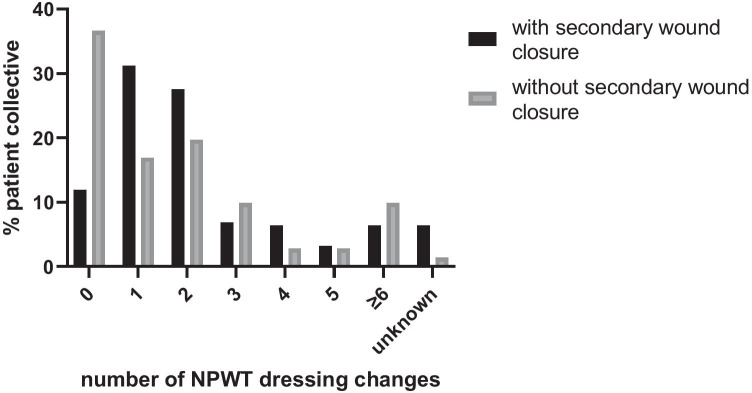
Number of NPWT dressing changes

Most patients (133 patients, 67.3%) received NPWT during the treatment of SSI in the postoperative period following a scheduled operative procedure compared to 32.7% of patients (65 patients), who received NPWT during the initial operation at our institution. The latter patients were mostly transferred to our hospital due to postoperative complications and, thus, received NPWT during the first operation at our hospital. On average, the NPWT lasted 10.5 ± 6.7 days with 2.1 ± 1.9 dressing changes, equaling a change every 5.1 days (Fig. 2).

### Perioperative data

The average length of hospital stay was 29.4 ± 18.8 days, and most patients had an intensive care unit (ICU) stay (60.4%; average duration: 12.6 ± 14.4 days). Of all the patients, 42.5% were treated on an intermediate care (IMC) unit, which lasted 6.2 ± 5.2 days. In terms of disposition, most patients were discharged home (65.7%), followed by a transfer to a rehabilitation center (19.2%). The in-house mortality of the patients with secondary skin closure after NPWT was 1.0% (Table 3).

**Table 3:** Discharge destination of patients after NPWT, with or without secondary skin closure other: transfer to different hospital, short-term care, geriatric care etc.

Discharge / transfer	With secondary skin closure (*n* = 198)	Without secondary skin closure (*n* = 67)
Into home environment	65.7% (130)	40.3% (27)
Rehabilitation facility	19.2% (38)	11.9% (8)
Death	1.0% (2)	29.9% (20)
Connection welfare treatment	2.5% (5)	5.9% (4)
Other	11.6% (23)	11.9% (8)

### Patient follow-up and wound characteristics

At the time of patient discharge, 37.2% of wounds after secondary skin closure had healed completely (Table 4). No detailed wound description was documented in a high number of patients (38.8%) at the time of discharge from the hospital. Two patients died during the hospitalization unrelated to the wound treatment and were not included in the analysis. In 7.7% of patients with secondary skin closure, a recurrent wound infection developed and required reopening the wound for a second time. Comparing this subgroup of patients who developed recurrent wound infections to those in our cohort who did not, some clear differences were found which reflect known risk factors for surgical site infections. Among those with recurrent infections, the rate of smoking was twice as high compared to those without recurrent infections (37.5 versus 18.2%, *p* = 0.06), a higher number were taking immunosuppressive medication (25.0 versus 14.0%, *p* = 0.23) or anticoagulation (50.0 versus 31.9%, *p* = 0.14), and the average BMI was higher (32.9 versus 28.9 kg/m^2^, *p* = 0.063).

**Table 4:** Comparison of wound conditions at the time of patient discharge

Wound condition	With secondary skin closure (*n* = 196)	Without secondary skin closure (*n* = 47)
Unknown	38.8% (76)	76.6% (36)
Completely healed	37.2% (73)	10.6% (5)
New wound infection	7.7% (15)	0%
Small wound dehiscence	6.6% (13)	0%
Persisting secretion of wound fluids	5.6% (11)	0%
Additional Prevena Vac treatment	1.5% (3)	0%
Partial wound closure	1.0% (2)	0%
Consolidating wound	1.5% (3)	6.5% (3)
Enterocutaneous fistula	0%	2.2% (1)
Second NPWT with skin closure	0%	4.3% (2)

We then followed these patients as outpatients for up to 2 years until April 2018 (Table 5). One additional patient died after discharge and was not included in this analysis. In 62.1% of cases, the wound had healed completely and the rate of persisting or new wound defect was low (4.6%). A total of 8.2% of patients developed an incisional hernia. However, 37 patients (19.0%) were not followed as outpatients at our institution; thus, we have incomplete information on the long-term results following secondary skin closure.

**Table 5:** Comparison of wound conditions during the post-discharge follow-up period

Wound condition	With secondary skin closure (*n* = 195)	Without secondary skin closure (*n* = 44)
Healed	62.1% (121)	43.2% (19)
Incisional hernia	8.2% (16)	13.6% (6)
Persisting or new wound defect	4.6% (9)	6.8% (3)
Enterocutaneous fistula	0.5% (1)	2.3% (1)
Divers (seroma, granuloma, etc.)	5.6% (11)	0%
Unknown	19.0% (37)	34.1% (15)

### Comparison to patients with NPWT followed by open wound treatment

During the analyzed hospitalization period, 67 patients treated with a NPWT did not receive a secondary skin closure but were continued on open wound treatment. We used this patient cohort as a reference group and compared patient characteristics, such as length of stay, wound status, and complications to those patients receiving a secondary skin closure.

### Patient characteristics, surgery, wound therapy, and hospital data

**Fig. 3:**
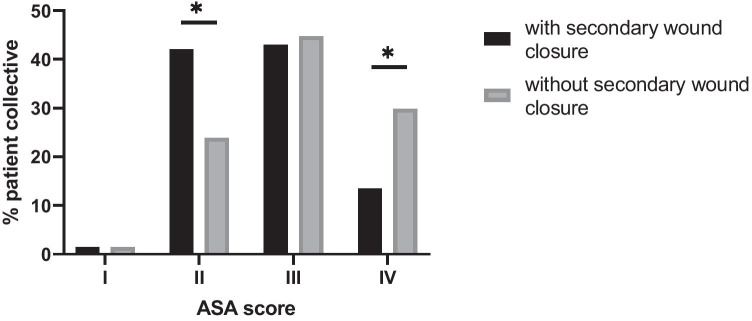
Comparison of ASA in patients with open wound therapy vs. secondary wound closure

There was no significant difference in gender distribution, BMI, use of chronic medication, tobacco use, or number of previous abdominal surgeries (Table 1 and Fig. 1). However, when looking at the ASA score, significant differences were found. A majority of the patients in the group without secondary skin closure were classified in the ASA III and IV stages (44.8/29.9%), whereas patients with secondary skin closure most commonly had ASA stages II and III (42.0/43.0%, Fig. 3).

The indication for surgery and the type of incision was comparable in both patient groups (Table 2). Similarly, the duration of NPWT and number of dressing changes did not differ (duration: secondary skin closure: 10.5 ± 6.7 days, open wound treatment: 10.9 ± 15.1 days; dressing changes: Fig. 2). When comparing the two patient groups regarding the duration of hospital stay, no difference was found (32.9 ± 23.7 days in patients with open wound therapy, *p* = 0.353). The duration of stay on the ICU or IMC wards in the group of patients without secondary skin closure was comparable as well (ICU: 66.2% for 14.4 ± 13.7 days, *p* = 0.318; IMC: 47.1% for 6.3 ± 5.6 days, *p* = 0.695). The length of hospital stay after application of the first NPWT dressing was 21.3 days in both patient groups, though the discharge destination in both patient groups showed a statistically significant difference (*p* < 0.001) with 65.7% patients discharged to home and 19.2% transferred to a rehabilitation facility in the skin closure group versus 40.3% patients returning home and 11.9% being transferred to a rehabilitation facility in the open wound treatment group (Table 3).

### Reasons for open wound treatment after NPWT instead of secondary wound closure

Of the 67 patients with open wound treatment after NPWT, a total of 20 patients died during hospitalization. Of those, 18 patients died during the NPWT and thus did not receive a secondary wound closure. Two patients died during the hospitalization but had the negative pressure therapy removed prior due to other reasons (high fluid output and development of an enterocutaneous fistula). In 17.6% of cases, the wound condition was satisfactory, leading to a waiving of the secondary wound closure. Other main reasons for foregoing secondary wound closure were poorly perfused wounds with no formation of granulation tissue (5.9%), the patient’s will (4.4%), the development of an enterocutaneous fistula (4.4%), and repeated bleeding or high fluid output (7.3%). In the remaining 17.9% of patients, a continued NPWT was not feasible due to a variety of different reasons, such as exudative eczema, dehiscence of fascia, location of the wound, or repeated air leaks.

### Wound condition after discharge from the hospital and during follow-up

At the time of hospital discharge, detailed wound description was not documented in 76.6% of patients (Table 4). We then also looked at the wound condition for up to 2 years after discharge until April 2018 (Table 5). Three additional patients died after discharge and were not included in this analysis. In most cases, the wound had healed completely (*n* = 19, 43.2%) and in only 6.8% a persisting or new wound defect was documented. The rate of incisional hernias was higher in patients treated with open wound therapy compared to the group with a secondary skin closure; however, this was not statistically significant (13.6% vs. 8.2%, *p* = 0.26). Unfortunately, 37 patients (19.0%) were not followed as outpatients at our institution; thus, we have incomplete information on the long-term results following open wound treatment after NPWT.

## Discussion

In this study, we examined the immediate and long-term results of infected abdominal wounds treated with NPWT followed by secondary surgical skin closure and then referenced these outcomes to a separate group treated with NPWT followed by continued open wound treatment.

Secondary skin closure after NPWT has been attempted successfully in our institution for several years as we increasingly saw successful results. However, the lack of literature on secondary skin closure of infected wounds after NPWT preconditioning prompted us to retrospectively analyze our practice of secondary skin closure in 200 consecutive cases in which a secondary skin closure was attempted when the patient and wound condition appeared suitable for it. As a secondary skin closure bears several advantages to the patient, this treatment option has become the primary treatment choice at our institution. In our retrospective analysis, we see that patients who did not receive a secondary skin closure most likely had prohibiting factors for a secondary skin closure. Naturally, a comparison between these two groups is not feasible but the analysis of patient and wound characteristics might shed light on the following questions:

1. How successful was the treatment of SSIs with NPWT and secondary skin closure overall?
2. Are there risk factors for recurring SSIs following skin closure?
3. Which patient or wound characteristics resulted in unsuccessful secondary skin closure?
4. Does the secondary skin closure decrease the hospital length of stay?

First, we analyzed four main patient characteristics previously shown to negatively impact wound healing: BMI, tobacco use, use of chronic medication, and ASA score. Looking at the BMI, we noticed the average BMI in the overall patient cohort being higher than that of the German population (29.2 m/kg^2^ versus 26.0 m/kg^2^ in 2011 according to the German Federal Statistical Office, www.destatis.de). Being overweight can negatively impact the wound healing process. A study examining patients after gynecological and abdominal operations found a higher rate of postoperative wound infections in patients with a BMI over 30 m/kg^2^ [17]. Reasons for poor wound healing in adipose tissue include a reduced number of capillaries resulting in decreased blood and thus oxygen supply [18]. Studies have shown a protective effect of a postoperative NPWT in adipose patients [19, 20]. In line with previous results, we saw that the patients with a recurrent wound infection after secondary skin closure had an increased average BMI when compared to the entire patient cohort, emphasizing BMI as a risk factor for recurrent wound infection and failed secondary skin closure.

As tobacco is known to have a negative effect on wound healing [21, 22], we analyzed the percentage of smokers in our cohort. Every fifth patient in our study is admitted to being an active smoker, which matches the estimated average in Germany [23]. Similar to the BMI, we did notice a higher percentage of smokers in the patient group with a recurrent wound infection after secondary skin closure. This again emphasizes smoking as a risk factor for wound infections.

Next, we analyzed the type of chronic medication patients were taking, especially immunosuppressant drugs (in particular steroids), which have been shown to negatively influence wound healing [24, 25]. Our study, though limited by the sample size, showed that a NPWT with or without secondary skin closure can be attempted even in patients on immunosuppressants. However, in line with previous results, we found more patients on immunosuppressant medication in the cohort with a recurrent wound infection following secondary wound/skin closure. Although anticoagulant therapy increases potential bleeding complications [26, 27], only two patients in our study required removal of NPWT due to bleeding complications. Thus, anticoagulant therapy does not seem to negatively influence the success of NPWT.

Looking at the ASA score, we found significantly more patients with an ASA stage IV in the group without secondary skin closure (29.9% versus 13.5%, *p* = 0.007), indicating that patients in the reference group had a higher risk of perioperative complications [28]. The NPWT and open wound treatment does not require anesthesia, whereas secondary skin closure requires anesthesia in most cases. This potential selection bias might very well have contributed to who was considered a candidate for secondary skin closure as well as the higher in-house mortality rate in this retrospective analysis.

Second, we analyzed the duration of the NPWT and the number of dressing changes prior to secondary skin closure or open wound treatment. Our results did not show a difference in either patient group (duration 10.9 and 10.5 days, without and with secondary skin closure respectively and with 2.1 dressing changes in both groups). Although NPWT manufacturers recommend dressing changes every 48 to 72 h [16], many studies have shown good results with a longer interval between dressing changes [29–31], which is in line with our results showing a good wound healing with a dressing change on average every 5 days. Less dressing changes save time and health care costs. A study from Switzerland compared the cost of a conventional dressing, which must be changed at least once daily and a NPWT with dressing changes every 3 days and found a significant cost reduction when applying the NPWT [32]. However, the evidence level of a potential reduction in health care cost by using NPWT is low, and comparisons with newer wound dressings requiring less frequent changes are lacking.

Third, we compared the operative and perioperative information of the two patient groups. When looking at the urgency, type of surgery, and incision site, we did not find a significant difference between the two patient groups. The length of hospital stay as well as the length of ICU and IMC stay after NPWT in patients with or without secondary skin closure was also similar. Though negative pressure therapy for infected wounds has been shown to promote wound healing and has advantages over conventional open wound treatment, it is a lengthy therapy [33]. Secondary skin closure after such a therapy might shorten overall treatment time and could have effects on the overall length of hospital stay. In our study, however, the length of hospital stay was comparable in both patient groups. There was a nonsignificant trend to a longer stay in the ICU or IMC in patients without secondary skin closure, which might be due to the higher ASA stage found in this patient group.

Lastly, and most importantly, we looked at the wound conditions and development of hernias after negative pressure therapy with and without secondary skin closure. In our study, at the time of hospital discharge, substantially more wounds after secondary skin closure had healed completely compared to patients with open wound treatment (37.2% versus 10.6%). Though in many cases the wound condition was not explicitly documented, one can assume a healed or at least noncomplicated wound condition in these cases, as problems or complications would be mentioned in discharge notes. During follow-up, the number of healed wounds increased and a persistent wound infection was documented in only 4.6% and 6.8% of patients with secondary skin closure or open wound treatment, respectively. One published case report showed a successful secondary skin closure after negative pressure therapy in an infected wound after spinal surgery in a previously radiated field. The secondary skin closure was performed 14 days after negative pressure therapy. During the 3-year follow-up, no additional wound infection arose [34]. Our study emphasizes this finding that secondary skin closure following negative pressure therapy is feasible. More studies, however, have looked at negative pressure therapy with instillation (delivery of topical wound solution in combination with negative pressure therapy) or closed-incision negative pressure therapy. Two retrospective studies compared negative pressure therapy with and without instillation and found that the secondary skin closure of lower limb wounds treated with negative pressure therapy with instillation was conducted significantly earlier [35, 36]. A meta-analysis comparing all studies on negative pressure therapy for closed surgical incisions (i.e. Prevena system) found a reduction in surgical site infections by approximately 40% compared to conventional wound treatment [37]. One study used the Prevena system on perineal wounds after neoadjuvant radio-chemotherapy followed by abdominoperineal resection of the rectum, which are known to result in wound healing disorders in up to 40% of cases. Similarly, this study was able to show a benefit of the Prevena system with five of the six patients (83%), experiencing a complication-free healing of the perineal wound [38]. This emphasizes the potential benefits of different types of negative pressure therapy in surgical incisions.

An additional and common complication after laparotomy is the development of an incisional hernia. Previous studies have reported an incidence ranging from 5 to 20% [39–41]. It has also been shown that negative pressure therapy promotes wound contraction, which can lead to a better secondary wound closure [42, 43]. In our study, incisional hernias were found slightly, yet not significantly, more often in the patient group with open wound treatment after 2 years of follow-up (8.2 versus 13.6%). This occurrence, however, is well within the previously published range, suggesting that secondary skin closure does not increase the risk of an incisional hernia.

In conclusion, secondary surgical skin closure following initial NPWT is largely successful and results in a very low rate of recurrent surgical site infection. Due to the retrospective nature of this study, we were unable to assess quality of life and the actual reduction in health care costs which will be evaluated in ongoing analyses. Furthermore, as a secondary skin closure was attempted when the patient and wound condition appeared suitable for it, a comparison to patients who did not receive a secondary skin closure is not possible due to prohibiting factors for a secondary skin closure. However, we feel that the advantages of a successful secondary skin closure in terms of quality of life and cost reduction appear self-evident. Thus, secondary skin closure is a valid alternative to the standard open wound treatment with the advantage of completed skin closure at the time of hospital discharge, and therefore reduced requirement for outpatient wound management.
